# Advanced utilization of motion decomposition and visualization technology in gymnastics education

**DOI:** 10.3389/fpsyg.2026.1794257

**Published:** 2026-03-20

**Authors:** Hechong Yang, Guangxin Cheng, Shuaichao Cheng

**Affiliations:** 1 School of Physical Education, Shandong University of Technology, Zibo, China; 2 School of Sports, Southwest University, Chongqing, China; 3 School of Physical Education, Shaoyang University, Shaoyang, China

**Keywords:** decomposition, digital education, gymnastics, instruction, movement, pedagogical reform, technology, visualization

## Abstract

With the ongoing advancements in higher education physical education reforms, gymnastics courses—a crucial element of sports education—necessitate the use of enhanced and new pedagogical approaches. Conventional gymnastics education encounters restrictions stemming from intricate motions, significant learning difficulties, and clear demonstration constraints, frequently resulting in students struggling to comprehend and master technical skills. To address these limitations, the present study integrates movement decomposition methodology with multiple visualization technologies to construct a structured, feedback-oriented instructional framework. Recent advancements in information technology within physical education have facilitated the development of visualization techniques in educational environments, providing innovative methods and tools for gymnastics training. This research focuses on the action decomposition teaching technique, incorporating visualization technologies including slow-motion replays, motion trajectory overlays, 3D motion models, and synchronous comparative analysis. It delineates a four-stage pedagogical model—“decomposition-visualization-practice-correction”—which differs from prior single-method approaches by systematically embedding real-time visual feedback at every stage of skill acquisition, and substantiates its efficacy through instructional trials and case analyses. Research findings demonstrate that visualization technology markedly enhances students’ cognitive comprehension of movement structures, elevates the quality of movement execution, boosts classroom engagement and participation, and cultivates students’ self-correction skills, thus realizing a significant innovation in gymnastics teaching methodologies. This study offers a pragmatic application demonstration and research foundation for the transformation of gymnastics instruction in higher education institutions.

## 1 Introduction

Gymnastics training is an essential element of physical education in higher education, significantly influencing students’ development of fundamental physical fitness, body control, and motor abilities (Podstawski et al., 2020). Gymnastic movements are distinguished by intricate structures, elevated technical requirements, and exact rhythmic performance (Sastre-Munar et al., 2022; Esteban-García et al., 2021). Students frequently face obstacles like ambiguous movement understanding, difficulty in comprehending essential components, and challenges in constructing mental imagery of the motions during the learning process (Colyer et al., 2018; Yeadon and Pain, 2023). Conventional pedagogical approaches, predominantly dependent on verbal elucidations and instructor demonstrations, exhibit distinct limitations: the efficacy of demonstrations is hindered by viewing angles, velocity, and students’ observational capacities, complicating the comprehensive presentation of movement intricacies (Mayer, 2024; Sweller et al., 2019); instructors encounter challenges in delivering individualized corrections for each learner (Herrebrøden et al., 2024); and students are deprived of efficient real-time feedback during practice (Richlan et al., 2023; Soltani and Morice, 2020). Consequently, the application of innovative technological methods to alleviate the challenges of learning gymnastics techniques, augment instructional efficacy, and elevate the quality of movement proficiency is a pressing concern in the ongoing reform of gymnastics education in higher academia (Pastel et al., 2023; Li, 2022).

In recent years, the profound integration of information technology with physical education has resulted in the extensive application of visualization techniques in educational contexts (Appelbaum and Erickson, 2018; Fábrica et al., 2019; Mödinger et al., 2022). In sports courses, tools like video replays, motion trajectory overlays, and 3D model projections have become indispensable resources (Post et al., 2016; Otte et al., 2020). Visualization technology transcends the temporal and spatial constraints of conventional displays (Özdemir and Seçkin, 2024). Utilizing slow-motion replays, action route annotations, and critical point magnifications, it offers pupils comprehensive and detailed movement information, hence enhancing the development of movement imagery (Zhang et al., 2021; Sun et al., 2017). The movement decomposition teaching method, a traditional technique in gymnastics training, reduces learning obstacles by segmenting intricate actions into digestible sub-movements, enabling pupils to progressively assemble the complete movement structure (Fiorella and Mayer, 2016). Combining movement decomposition with visualization technology to create a cohesive, feedback-oriented teaching method has the potential to greatly improve students’ comprehension of movement principles and structure, streamline learning paths, and reduce skill acquisition durations (Tai et al., 2023).

While both local and foreign research has examined gymnastics pedagogy and visualization technology, the majority of studies concentrate on the implementation of individual instruction approaches (Fan et al., 2025; Lou and Jaeggi, 2020). Specifically, existing studies typically apply either movement decomposition or visualization technology in isolation: decomposition-based approaches (e.g., hierarchical task analysis) focus on segmenting movement structure but provide limited real-time visual feedback, while visualization-only interventions (e.g., video modeling, VR training) improve perceptual learning but lack a systematic progression framework linking cognitive understanding to motor consolidation. There is a deficiency of comprehensive research on the amalgamation of movement decomposition methodologies with visualization technologies, and no broadly applicable systematic teaching model has been developed (Zhao et al., 2023). The four-stage “Decomposition-Visualization-Practice-Error Correction” model proposed in the present study addresses this gap by uniquely coupling structured movement segmentation with multi-modal visual feedback at each stage, creating a closed-loop learning cycle that neither decomposition-only nor visualization-only approaches can provide. Consequently, in light of the continuous reform in physical education and the progression of information technology applications, it is imperative to develop a novel gymnastics teaching model grounded in movement decomposition and visualization technology, and to assess its feasibility and efficacy through pedagogical practice. This study seeks to investigate particular pedagogical paths that amalgamate both methodologies, formulating a gymnastics skill instruction process of decomposition visualization practice correction. It aims to deliver effective pedagogical reform strategies for university gymnastics courses and furnish theoretical and practical foundations for the digitalization of physical education instruction.

## 2 Theoretical framework

Acquiring gymnastic movements is a multifaceted process that encompasses perception, cognition, movement planning, and execution, with visual information being crucial for comprehending motions and honing skills (Karunakaran et al., 2024). Motor learning theory posits that learners must initially create a mental representation of the movement using external signals, subsequently develop stable movement patterns through repetitive practice, and continuously refine these patterns based on feedback (Sepp et al., 2019; Kirk and MacPhail, 2002). Conventional pedagogical approaches frequently depend on restricted visual stimuli, hindering students’ ability to comprehend the temporal dynamics and force application of movements effectively (Dyson et al., 2004). Visualization technology, nonetheless, offers consistent, transparent, and reproducible movement data. This allows learners to have sufficient visual stimulation during the cognitive phase of skill acquisition, therefore improving movement understanding and expediting the automation of motor skills (Chiviacowsky and Drews, 2016).

Constructivist learning theory highlights active exploration and the creation of meaning in genuine circumstances (Wulf and Lewthwaite, 2016). Gymnastics motions, being highly programmed motor abilities, are challenging for children to fully grasp through teacher explanations and demonstrations alone (Collins et al., 2015). Visualization technology fosters more immersive and genuine educational experiences. Students can engage in the observation, analysis, and reflection on movement processes via slow-motion replays, motion path annotations, and synchronized video comparisons (Araújo and Davids, 2016). This recurrent process of refining and validation enables the development of movement knowledge (Woods et al., 2020). Moreover, the movement decomposition technique disaggregates intricate actions into essential sub-tasks, rendering learning processes more tangible and controllable (Williams and Hodges, 2005). When integrated with visualization technology, students have immediate and accurate visual feedback at each sub-step, enhancing the comprehension of movement significance.

Multimodal learning theory establishes the psychological basis for utilizing visualization technology in gymnastics education. Mayer’s multimodal cognitive theory posits that learners assimilate information via dual visual and verbal channels, resulting in enhanced understanding of intricate material in settings that combine imagery and text. Gymnastic motions have considerable spatio-temporal qualities, rendering their dynamic structures challenging to articulate just through linguistic explanations. Multimedia forms, including photos, videos, animations, and 3D models, facilitate more effective information processing for pupils within constrained cognitive resources. When these multimedia tools augment the instructor’s verbal explanations, they diminish cognitive load, improve the efficiency of action information retrieval, and aid in the development of comprehensive and precise motor memory representations.

Action learning theory, constructivist learning theory, and multimedia learning theory collectively establish the theoretical framework for incorporating movement deconstruction and visualization approaches into gymnastics education. These theories offer a logical basis for pedagogical innovation across various dimensions such as movement formation, cognitive construction in learners, and information processing mechanisms, clarifying the essential role of visualization technology and decomposed instruction in improving gymnastics skill acquisition.

## 3 Methodologies of research

This study examines the efficacy of motion decomposition and visualization technologies in gymnastics education, utilizing several research methodologies to guarantee scientific rigor and reliability. The literature review establishes the theoretical framework. This study analyzes domestic and international literature on gymnastics pedagogy, visualization technology, and motor learning theory to outline the evolution of instructional reform, pinpoint research opportunities, and furnish evidence for developing teaching models (Ste-Marie et al., 2016).

A teaching experiment was conducted to assess the efficacy of the new educational model. Two natural courses from a university gymnastics course were chosen as participants, constituting an experimental group and a control group. The two natural classes were not randomly assigned; however, pre-test comparisons confirmed no statistically significant differences between groups in age, gender ratio, or baseline gymnastics proficiency (independent samples *t*-test; all *p* > 0.05), thereby supporting the comparability of the two groups prior to the intervention. A 6-week instructional intervention was conducted, emphasizing comparative experimentation with fundamental motions including handstands and rolls. Questionnaire surveys and classroom observation methods were utilized to thoroughly assess student learning performance and experiences (Wang and Wang, 2024).

Questionnaires evaluated subjective experiences across domains such as learning interest, movement understanding, and self-correction capability. Skill proficiency was evaluated using a standardized 100-point rubric, and two certified gymnastics instructors independently scored each student’s performance; the inter-rater reliability between the two evaluators was high (Intraclass Correlation Coefficient, ICC = 0.92; 95% CI: 0.88–0.95), confirming the objectivity and consistency of the assessment process. Scores were averaged across the two raters for subsequent analysis. Classroom observations recorded performance, prevalent errors, and engagement levels, augmented by teaching logs for further analysis (Jastrow et al., 2022). Furthermore, interviews obtained genuine input from students and teachers to enhance the research data. Ultimately, statistical analysis examined experimental data to assess variations in the efficacy of instructional models.

All between-group comparisons of post-test scores were conducted using independent samples *t*-tests. Within-group pre-to-post changes were evaluated using paired-samples *t*-tests. Effect sizes were calculated using Cohen’s d. The significance threshold was set at α = 0.05. All analyses were performed using SPSS 26.0.

## 4 Instructional design for movement decomposition and visualization technology

Gymnastics movements exhibit intricate frameworks, extensive technical specifications, and significant requirements for bodily coordination (Potdevin et al., 2018; Fábrica et al., 2019). Students may encounter ineffective skill learning owing to inadequate comprehension or ambiguous movement visualization. This study enhances standard movement decomposition teaching approaches by integrating several visualization technologies to provide an instructional design focused on cognitive clarity, process visualization, structured practice, and accurate error correction (Otte et al., 2020). This design is methodically developed across four dimensions: instructional objectives, movement decomposition tactics, visualization application techniques, and integrated process design (Mödinger et al., 2022).

### 4.1 Instructional goal formulation: integrated advancement of cognitive, skill, and affective domains

**FIGURE 1:**
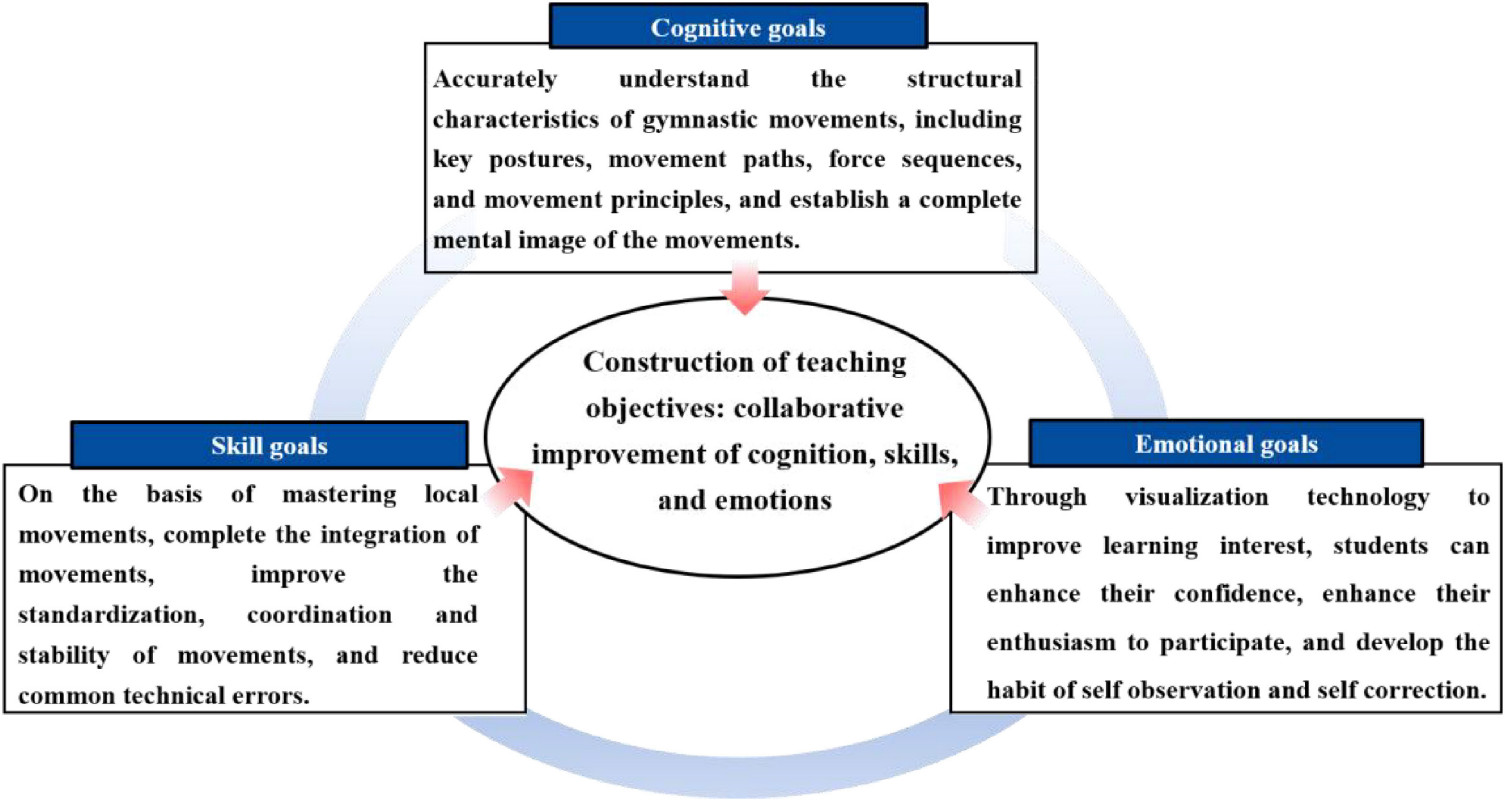
Construction of teaching objectives.

This instructional design, informed by the “Guidelines for Physical Education Curriculum in Higher Education Institutions” and the practical attributes of gymnastics courses, includes three kinds of objectives (Figure 1). It underscores the importance of acquiring technical skills while aligning with the contemporary philosophy of physical education that promotes integrated cognitive, skill-based, and affective development.

### 4.2 Gymnastics movement decomposition strategy: mitigating complexity, emphasizing essential components

Movement decomposition is the predominant technical teaching method utilized in gymnastics instruction (Potdevin et al., 2018; Huang et al., 2023). The fundamental idea entails deconstructing intricate movements into smaller, action-compliant elements, allowing trainees to progressively grasp essential components. This research enhances decomposition tactics across three dimensions: movement structure patterns, biomechanical characteristics, and the physical and mental development levels of pupils, as illustrated in Table 1.

**TABLE 1:** Breakdown strategy for gymnastics movements.

Decomposition dimension	Content
Action structure-based hierarchical decomposition	Deconstruct the movement structure into segments of “preparation—transition—key point—conclusion,” facilitating students’ understanding of the technical role of each component within the overarching framework. In instructing forward rolls, segment the technique into five phases: squat preparation, hand support, tucking the head and chest, rolling the back, and culminating in a standing position. Each phase relates to specific technological prerequisites.
Key point decomposition based on kinematics	Concentrate on deconstructing fundamental components such as force orientation, support point transitions, and center-of-gravity relocation to facilitate students’ understanding of movement mechanics. When instructing on handstands, highlight three essential technical aspects: “stable hand support,” “hip-driven upward lift,” and “vertical alignment of the center of gravity.” This method allows pupils to grasp the fundamental nature of the movement during practice.
Personalized breakdown based on learning difficulties	Students face diverse obstacles in motor skill acquisition, requiring adaptability in instructional design. Educators ought to provide tailored, incremental workouts addressing specific challenges—such as inadequate shoulder strength or deficient core stability—to facilitate differentiated instruction.

### 4.3 Utilization techniques of visualization technology: augmenting movement imagery and immediate feedback

**FIGURE 2:**
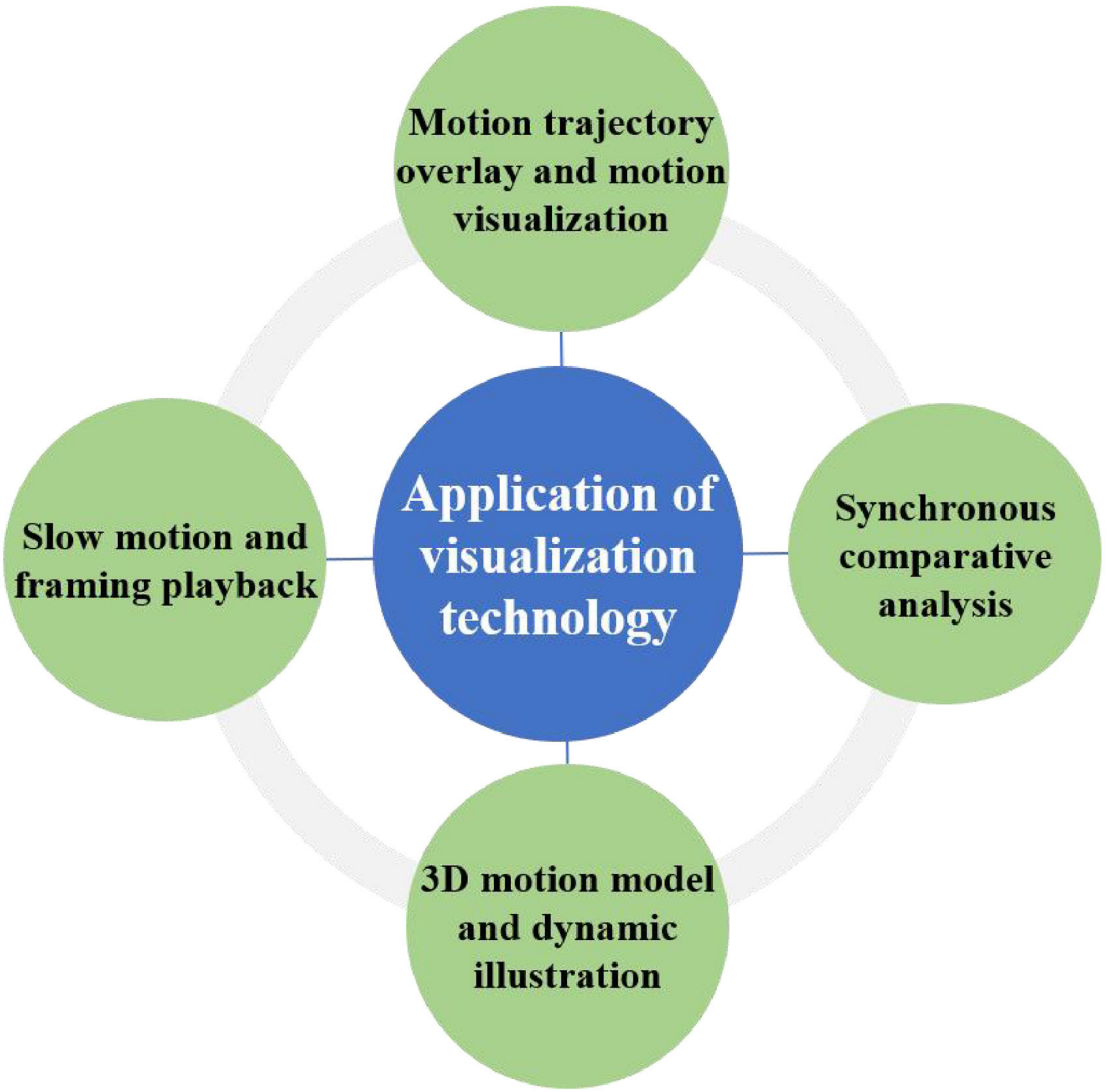
Application mode of visualization technology.

This study employed advanced visualization technologies to address the constraints of conventional demonstration methods in gymnastics education (Otte et al., 2020; Starzak et al., 2022). The specific hardware and software configurations used were as follows: video recording was performed using Sony AX700 4K camcorders and Apple iPhone 14 Pro smartphones at 120 frames per second for slow-motion capture; motion trajectory overlay and center-of-gravity visualization were implemented using Kinovea (v0.9.5); synchronized comparative analysis was conducted using Dartfish TeamPro (v10.0); three-dimensional motion modeling and dynamic diagramming were created using Blender (v3.6); and classroom playback was delivered via a 75-inch 4K interactive touchscreen panel. These tools were integrated to enhance students’ movement understanding, learning efficiency, and self-correction across multiple dimensions (Figure 2).

Initially, slow-motion and frame-by-frame replay technology constitute a fundamental element of this research. By recording student movements with mobile phones or professional cameras and replaying them in slow motion, students can distinctly observe details of the actions, including the timing of force application, alterations in posture, and adjustments in dynamic joint angles. The continued use of frame-by-frame replay disaggregates the movement sequence into individual static images, allowing students to meticulously examine each pivotal moment and thereby facilitate the identification and rectification of faults. This technology is very proficient for intricate or challenging movements, assisting students in surmounting prevalent comprehension obstacles in conventional teaching methods. Secondly, motion trajectory overlay and center-of-gravity visualization technology utilizes motion analysis software to superimpose students’ movement trajectories as trajectory lines onto video footage. This enables students to visually monitor alterations in body weight distribution and variations in limb trajectories. For example, while mastering a side handstand, trainees may distinctly recognize their body’s deviation spots and make necessary adjustments, thereby improving movement precision and coordination. This device offers spatial awareness feedback via visual signals, a capability unattainable by conventional teaching approaches. Moreover, synchronized comparative analysis technology enables the playback of students’ motions in conjunction with standard demonstration film, guaranteeing precise alignment in both velocity and framing. This comparison enables pupils to more readily discern discrepancies in their motions, so enhancing self-monitoring capabilities and fostering self-regulation and learning. This immediate feedback allows students to implement specific modifications during each practice session. 3D motion modeling and dynamic diagramming technology employ three-dimensional models or animations to depict the relative placement of body parts during motions, the directions of force application, and the logical framework of actions. This technology is especially advantageous for actions where teachers find it challenging to explain from various perspectives, such as center-of-gravity management during handstands or trunk flexion/extension variations during rolls. Multi-angle rotating 3D models provide students with profound insights into the principles and requirements of motions from diverse perspectives, thereby considerably augmenting their spatial awareness.

### 4.4 Integrated process design: the “decomposition-visualization-practice-error correction” instructional model

**FIGURE 3:**
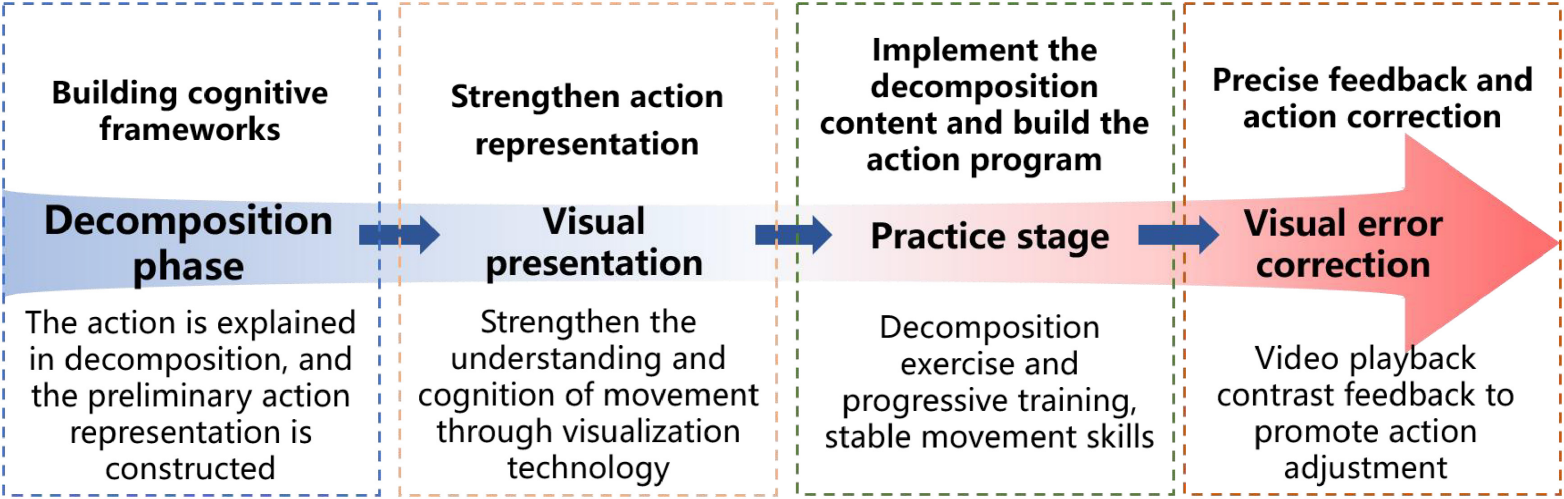
Teaching mode of “decomposition visualization practice error correction.”

This study developed a four-stage educational process “Decomposition-Visualization-Practice-Error Correction” to effectively integrate decomposition teaching with visualization technology, as depicted in Figure 3, thereby facilitating the efficient mastering of gymnastics abilities. This paradigm adheres to the cognitive principles of gymnastics movement acquisition, advancing from the establishment of a cognitive framework to the consolidation of skills. Through a cycle of observation, imitation, practice, and feedback, it perpetually enhances students’ understanding of movement and skill development.

The breakdown stage functions as the initial educational phase. Instructors employ movement decomposition techniques and decomposed motion movies to assist students in forming initial mental representations of the motions. Students primarily comprehend the logical progression of motions by analyzing their structural elements and critical moments, establishing a cognitive foundation for future learning. The visualization phase utilizes slow-motion replays, frame-by-frame analysis, and 3D modeling to illustrate each deconstructed part, highlighting essential movement points and safety implications. Clear and intuitive visual information diminishes misconceptions, improves cognitive precision, and assists pupils in constructing accurate mental representations of movements. In the practice phase, students do each segmented movement sequentially, adhering to video demonstrations, while instructors offer specific guidance based on the visual material. This phase utilizes a progressive training methodology that merges individual practice with integrated drills. Students sequentially master each movement segment, progressively assembling the entire movement sequence. Through ongoing practice refining, they finally stabilize motor skill formation. The visual error repair phase employs video replays and synchronized comparative analysis to assist students in identifying and rectifying movement discrepancies. Students actively modify and improve their performance by identifying gaps between their execution and the standard movements, therefore boosting movement quality. This visual real-time feedback system enhances students’ identification of movement discrepancies and fortifies their self-regulation and error-correction skills, becoming a vital component in enhancing educational results.

## 5 Instructional experiment and impact assessment

This study utilized a controlled experimental method to assess the practical usefulness of motion decomposition integrated with visualization technology in gymnastics training, methodically validating the feasibility and efficacy of the new teaching model (Lamošová and Kyselovičová, 2022; Post et al., 2016). The experiment performed an extensive analysis focused on multidimensional indicators such as students’ proficiency in motion, learning engagement, self-correction skills, and classroom participation, with the objective of thoroughly illustrating the effects of the teaching reform (Ste-Marie et al., 2016; My et al., 2022; Wulf and Lewthwaite, 2016).

### 5.1 Experimental design

The research involved 80 students from two natural classes in a university gymnastics course as participants. The participants were allocated into an experimental group and a control group, with each group consisting of 40 pupils. As noted in Section 3, the allocation was based on existing natural class groupings rather than random assignment; pre-test equivalence was confirmed statistically (all *p* > 0.05). The experimental duration lasted 6 weeks, encompassing essential actions like handstands, forward rolls, and side handstands (Post et al., 2016). The experimental group implemented a novel pedagogical model integrating “movement decomposition + visualization technology,” whereas the control group adhered to the traditional “explanation + demonstration + practice” method. The experiment occurred in three stages: Phase 1 (Pre-test): Both groups participated in gymnastics fundamentals evaluations and learning attitude surveys to confirm baseline equivalence. Phase 2 (Instructional Implementation): Each group executed the same instructional content utilizing different pedagogical methodologies. Phase 3 (Post-test): Comparative assessments evaluated skill performance, learning engagement, and self-correction capabilities to analyze instructional outcomes.

### 5.2 Experimental metrics and data acquisition

The study established the following indicators to thoroughly assess instructional effectiveness:

#### 5.2.1 Skill proficiency indicators

In accordance with gymnastics curriculum standards, student performance was evaluated using a standardized 100-point rubric assessing four dimensions: (1) movement uniformity (25 points): consistency of rhythm and sequential coordination across the full movement; (2) execution quality (30 points): accuracy of limb positioning, joint angles, and force application at key technical points; (3) stability (25 points): maintenance of balance and postural control throughout the movement; and (4) whole-body coordination (20 points): integration of upper and lower limb movements and core engagement. Scoring descriptors were pre-established and reviewed by the two certified evaluators prior to data collection to ensure shared interpretive standards. Two certified instructors separately evaluated each student, with scores averaged to improve objectivity. Inter-rater reliability was confirmed with ICC = 0.92 (95% CI: 0.88–0.95). The skill scores for the experimental and control groups, both pre-test and post-test, are presented in Table 2.

**TABLE 2:** Comparison of motor skill scores between the experimental and control groups before and after intervention.

Group	Pre-test (M ± SD)	Post-test (M ± SD)	Increase	*t*	*p*	Cohen’s *d*	95% CI
Experimental group (*n* = 40)	61.8 ± 5.2	81.4 ± 4.8	+19.6	8.34	<0.001	1.87	[6.4, 10.8]
Control group (*n* = 40)	62.1 ± 5.4	72.8 ± 5.1	+10.7				

SD, standard deviation. *t*-test compares post-test scores between groups. 95% CI refers to the confidence interval for the mean difference between post-test scores.

#### 5.2.2 Learning interest indicators

A five-point Likert scale questionnaire was utilized, encompassing variables such as learning motivation, activity interest, classroom satisfaction, self-confidence, and commitment to continued practice. The specific questionnaire items administered to students were as follows: (1) Learning Motivation—“I am motivated to learn and improve my gymnastics skills in this course”; (2) Interest in Motor Learning—“I find the process of learning new gymnastics movements engaging and enjoyable”; (3) Classroom Enjoyment—“I feel satisfied with the overall atmosphere and content of the gymnastics class”; (4) Self-confidence—“I feel confident in my ability to correctly perform the gymnastics movements taught in class”; (5) Willingness to Practice Consistently—“I am willing to practice gymnastics movements outside of class time to further improve my skills.” Each item was rated on a scale from 1 (Strongly Disagree) to 5 (Strongly Agree). Cronbach’s α for the full scale was 0.87, indicating good internal consistency. Questionnaire findings are displayed in Table 3.

**TABLE 3:** Results of the Likert five-point scale questionnaire.

Dimension	Pre-test Exp. (M ± SD)	Post-test Exp. (M ± SD)	Pre-test Ctrl. (M ± SD)	Post-test Ctrl. (M ± SD)	*t*	*p*
Learning motivation	3.10 ± 0.52	4.25 ± 0.48	3.08 ± 0.54	3.40 ± 0.50	7.91	< 0.001
Interest in motor learning	3.05 ± 0.55	4.20 ± 0.51	3.01 ± 0.53	3.35 ± 0.49	8.16	< 0.001
Classroom enjoyment	3.20 ± 0.50	4.35 ± 0.46	3.18 ± 0.51	3.55 ± 0.47	7.44	< 0.001
Self-confidence	2.90 ± 0.58	4.10 ± 0.53	2.88 ± 0.57	3.20 ± 0.52	9.02	< 0.001
Willingness to practice	3.00 ± 0.54	4.28 ± 0.49	2.95 ± 0.56	3.38 ± 0.51	8.63	< 0.001
Overall average	3.05 ± 0.54	4.24 ± 0.49	3.02 ± 0.54	3.38 ± 0.50	8.28	< 0.001

#### 5.2.3 Self-correction ability metric

A thorough evaluation of students’ capacity to notice, analyze, and rectify incorrect motions is performed using classroom observation records and semi-structured interviews. The semi-structured interview guide used with students consisted of the following questions: (1) “When you reviewed your own movement video, were you able to identify specific technical errors? Please describe an example.”; (2) “Could you explain why you think that error occurred in terms of body position or force application?”; (3) “After identifying a problem, what specific adjustments did you make in subsequent practice sessions, and were they effective?” Responses were scored on a 10-point scale across three sub-dimensions: (a) ability to identify errors (0–4 points); (b) ability to analyze underlying causes (0–3 points); (c) ability to implement self-correction (0–3 points). This instrument was developed and validated by a panel of three experienced gymnastics educators prior to the study. Table 4 displays the scores for self-correction ability.

**TABLE 4:** Self-correction ability scores.

Group	Pre-test (M ± SD)	Post-test (M ± SDs )	Increase	*t*	*p*	Cohen’s *d*	95% CI
Experimental (*n* = 40)	4.2 ± 0.8	8.1 ± 0.7	+3.9	12.43	< 0.001	2.79	[1.8, 2.6]
Control (*n* = 40)	4.3 ± 0.9	6.0 ± 0.8	+1.7				

### 5.3 Examination of experimental outcomes

Experimental findings demonstrate that students in the experimental group attained markedly superior average scores in movement execution quality relative to the control group. Students in the experimental group exhibited enhanced body verticality, shoulder stability, and core control, particularly during handstand exercises. The between-group difference in post-test skill scores was statistically significant [*t*(78) = 8.34, *p* < 0.001, Cohen’s *d* = 1.87], indicating a large effect size. Movement quality scores enhanced by an average of 15–25%.

This result indicates that visualization technology, by illustrating distinct movement trajectories and essential reference points, markedly improves students’ comprehension of movement structure, facilitating the quicker establishment of stable movement patterns. The learning interest questionnaire indicated that students in the experimental group achieved significantly higher scores than those in the control group in areas including “curiosity regarding gymnastics movement acquisition,” “enjoyment in the classroom,” “readiness for independent practice,” and “confidence in executing movements.” The experimental group’s mean interest score rose by almost 20% relative to the pre-test, but the control group’s enhancement was below 5%. The multi-faceted, dynamic, and zoomable observation features of visual teaching technology significantly improved the learning experience, increasing student engagement.

Upon examining their movement recordings, students in the experimental group proactively detected difficulties such as trajectory deviations, inadequate support angles, and trunk instability, showcasing significantly improved comparison analytical skills. The between-group difference in self-correction ability post-test scores was also statistically significant (*t* = 12.43, *p* < 0.001, Cohen’s *d* = 2.79), representing a very large effect. In interviews, the majority of students indicated that visual error correction techniques were “more comprehensible regarding the problem” and “more intuitive than verbal explanations from teachers.” These findings indicate that visualization technology mitigates the abstract and challenging nature of conventional verbal corrections, hence augmenting students’ abilities for self-reflection and self-regulation. The statistical results indicate that the experimental group exhibited considerable enhancements in motor skill scores, learning interest, and self-correction abilities, with all increases markedly surpassing those of the control group. The experimental group exhibited an average score gain of 19.6 points, whereas the control group demonstrated an increase of 10.7 points in skill assessment. The experimental group’s average score in learning interest metrics increased by 1.19 points across five dimensions, contrasting significantly with the control group’s 0.36-point rise. By intuitively conveying movement details and augmenting classroom interaction, visual technology effectively encouraged students’ interest and engagement in learning. The experimental group had a 3.9-point enhancement in self-correction capacity, far surpassing the control group’s 1.7-point increase. Visualized recordings and realtime comparative analysis allowed students to autonomously recognize problems and modify movement tactics, greatly improving proactive learning and self-regulation skills. The experimental data unequivocally indicate that the “movement decomposition + visualization technology” teaching approach offers significant benefits in fostering the holistic development of students’ motor skills, hence offering substantial empirical evidence for gymnastics teaching reform.

## 6 Case study: visual reinforcement and movement decomposition in handstand instruction

The handstand, as a fundamental technique in gymnastics courses, necessitates considerable shoulder strength, core stability, and body balance from students, functioning as an essential element of movement control training (Zemková and Zapletalová, 2022). In traditional education, prevalent student challenges encompass inadequate arm support, inconsistent force distribution during leg elevation, difficulty in sustaining vertical alignment, and either the collapse or hyper-arching of the lower back. This study utilizes the handstand as a case study, employing movement decomposition tools alongside visualization techniques to develop a thorough teaching implementation framework (Potdevin et al., 2018; Kok et al., 2020).

Instruction commences with a hierarchical analysis of the handstand with the movement decomposition technique (Ste-Marie et al., 2016). The handstand is categorized into five essential steps based on its characteristics: preliminary posture, arm support establishment, leg elevation, vertical alignment maintenance, and controlled fall. Each phase distinctly delineates technical aspects. The preliminary phase highlights hand placement width and shoulder tension, the leg lift phase underscores hip engagement and core tightening, and the vertical hold phase concentrates on center of gravity alignment and body axis control. This methodical technique allows students to gradually acquire proficiency in essential actions while comprehending the overarching framework. Visual technology is utilized via slow-motion video demonstrations of instructor motions, enabling students to examine alterations in limb angles and adjustments in center of gravity during the leg lift. Moreover, motion path overlay technology annotates students’ practice films with movement trajectory lines, effectively emphasizing difficulties such as body deviation locations and support misalignment. During the vertical hold phase, overlaid vertical reference lines allow pupils to accurately evaluate their posture against vertical standards, hence improving self-adjustment abilities. The synchronous comparison analysis technique enhances pupils’ movement cognition. Educators exhibit students’ actions in conjunction with conventional demonstrations concurrently. Through the observation of these discrepancies, students actively discern fault types—such as inconsistent lifting velocity, deviations in hip angle, or shoulder collapse. By means of continual comparisons, students progressively cultivate a conceptual comprehension of optimal movement states, facilitating more precise corrections in future practice (Ste-Marie et al., 2016).

Following several teaching cycles, pupils’ handstand performance exhibited significant enhancement. The majority rapidly acquired arm support stability, prolonged vertical body hold time, and improved core control. Instructor evaluations suggest that integrating movement analysis with visualization technology enhances instructional efficacy and promotes student independence, resulting in notable improvements in handstand training. This study illustrates the tangible benefits of amalgamating these two pedagogical approaches, providing a reproducible framework for instructing more intricate gymnastics maneuvers.

## 7 Discussion

This study illustrates that the profound integration of movement decomposition with visualization technology is essential for improving the efficacy of gymnastics education (Wulf and Lewthwaite, 2016; Zhang et al., 2025). Nonetheless, despite recognizing its benefits, additional discourse is required concerning pedagogical applicability, resource distribution, and sustainable development (Svennberg, 2017).

This methodology enhances teaching efficacy by addressing shortcomings in traditional training, including abstract movement concepts, insufficient demonstrations, and delayed feedback (Yang, 2025). The movement decomposition technique establishes a more organized learning process, allowing students to gradually acquire essential movement components (Wulf and Lewthwaite, 2016). Visualization technology substantially improves movement recognition precision and reduces the learning duration by augmenting visual input and delivering prompt feedback (Lyzenga and Previsic, 2021). This educational reform imposes greater requirements on teachers’ professional competencies. Instructors must not only exhibit proficient athletic skills and pedagogical expertise but also attain mastery in the operation of digital tools, hence introducing new hurdles for teacher training programs (Svennberg, 2017). Secondly, from a resource allocation standpoint, the implementation of visual instruction need technical assistance, encompassing filming apparatus, analytical software, and display devices. Although most colleges possess fundamental technical capabilities, the consistent application requires enhanced equipment configuration and the advancement of teaching environments (Ruiz-Ariza et al., 2018). For institutions with constrained resources, promoting this approach without incurring substantial expenditures remains a pivotal challenge for future adoption. Moreover, students may cultivate a propensity for technological reliance while utilizing visualization technologies, excessively relying on visual feedback and disregarding the enhancement of their own body awareness (Garn et al., 2019). Gymnastics training prioritizes internal perception and movement regulation; thus, instructors ought to limit the application of visualization technology in instruction to avoid supplanting the development of students’ bodily awareness (Lasda and Parker, 2016). This teaching technique is excellent for foundational gymnastics routines, but its relevance to more complicated gymnastics competitions necessitates additional experimental confirmation. Moreover, improvements in artificial intelligence and motion capture technologies may facilitate the future incorporation of intelligent motion recognition and real-time scoring systems into the educational framework, thereby creating a more robust technological support structure (Xing et al., 2022; Lyzenga and Previsic, 2021).

## Data Availability

The raw data supporting the conclusions of this article will be made available by the authors, without undue reservation.
